# Does Hsp60 Provide a Link between Mitochondrial Stress and Inflammation in Diabetes Mellitus?

**DOI:** 10.1155/2016/8017571

**Published:** 2016-07-12

**Authors:** Joshua Juwono, Ryan D. Martinus

**Affiliations:** School of Science, University of Waikato, Private Bag 3105, Hamilton 3240, New Zealand

## Abstract

The focus of this review is to summarise the known relationships between the expression of heat shock protein 60 (Hsp60) and its association with the pathogenesis of Type 1 and Type 2 diabetes mellitus. Hsp60 is a mitochondrial stress protein that is induced by mitochondrial impairment. It is known to be secreted from a number of cell types and circulating levels have been documented in both Types 1 and 2 diabetes mellitus patients. The biological significance of extracellular Hsp60, however, remains to be established. We will examine the links between Hsp60 and cellular anti- and proinflammatory processes and specifically address how Hsp60 appears to affect immune inflammation by at least two different mechanisms: as a ligand for innate immune receptors and as an antigen recognised by adaptive immune receptors. We will also look at the role of Hsp60 during immune cell activation in atherosclerosis, a significant risk factor during the pathogenesis of diabetes mellitus.

## 1. Introduction

Diabetes mellitus is a spectrum of metabolic disorders characterised by chronic hyperglycaemia and abnormalities within the metabolism of proteins, fats, and carbohydrates [1]. The two most common forms of diabetes are classified as Type 1 and 2. Type 1 also known as insulin-dependent diabetes (IDDM) is characterised by the autoimmune destruction of the *β*-cells of the pancreatic islets which ultimately results in loss of insulin production leading to hyperglycaemia [2]. On the other hand, Type 2 diabetes is linked to disorders of both insulin secretion and insulin action [3]. It is becoming increasingly clear that Type 2 diabetes is also associated with a progressive destruction of *β*-islet cells by autoimmune processes linked to inflammation [4]. However, the key triggers and molecular mechanisms responsible for the loss of *β*-cells have not yet been elucidated. Since mitochondria play a key role in the secretion of insulin from *β*-islet cells [5], in this review we will examine the role of the mitochondrial molecular stress protein Hsp60 in the pathogenesis of both Types 1 and 2 diabetes and the potential links between Hsp60 expression and inflammation.

## 2. Role of Mitochondrial Hsp60 in Types 1 and 2 Diabetes Mellitus

Heat shock protein 60 (Hsp60) is a molecular stress protein predominantly localised to the mitochondrion where it is known to play a role in the folding of proteins in the mitochondrial matrix. Hsp60 is upregulated in response to mitochondrial impairment [6] and is considered to be an indicator of mitochondrial stress. Interestingly, Hsp60 has also been shown to be secreted from a variety of mammalian cell types [7–9] and is known to be found at elevated levels in both Types 1 and 2 diabetes mellitus [10]. The physiological/pathological consequence of having elevated levels of Hsp60 in systemic circulation and the cell types responsible for secretion of Hsp60 into circulation in diabetes mellitus is not yet known.

For many years Hsp60 has been observed in nonobese (NOD) mouse model of diabetes and has been linked to play a role in the destruction of pancreatic *β*-islet cells caused by the spontaneous development of autoimmune T-lymphocytes [11]. Several epitopes have been identified to have significant anti-Hsp60 T-cells responses, but one in particular, p277 peptide, has shown the greatest anti-Hsp60 T-cells responses [12]. The p277 peptide of Hsp60 has been widely studied over the years; it is 24-amino acid peptide within amino acid residues 427–460 derived from monocytes human Hsp60 [13]. Hyperglycaemia and insulitis developed when standard strains of mice, not prone to spontaneous diabetes, were immunized with p277 covalently conjugated to a foreign immunogenic carrier molecule [14].

Interestingly, Hsp60 and p277 peptides can also lead to protection of *β*-cell functions [15, 16]. This protection is thought to be due to modulation of the autoimmune process responsible for *β*-cell destruction. Therapeutic vaccination with p277 has been documented to slow and inhibit the destruction of *β*-cells both in NOD mice and in humans. Administration of p277 has been shown to downregulate T-cell reactivity to *β*-cell antigens. This process is thought to be associated with a shift in cytokine profile from the proinflammatory T-helper 1 (Th1) phenotype to the anti-inflammatory T-helper-2 (Th2) phenotype. Thus, p277 has been shown to increase IL-4 and IL-10 secretion and decrease *γ*-IF secretion and is thought to be mediated by p277 binding to TLR2 receptors [17, 18].

Therefore, it is evident that Hsp60 is able to influence T-cell responses in two ways: as a ligand of toll-like receptor 2 signalling and as an antigen. But how can T-lymphocytes target Hsp60 (which is ubiquitous) and p227 epitope be involved with the destruction and protection of pancreatic *β*-cells? One explanation for this is molecular mimicry. Hsp60 and p277 could possibly mimic a tissue-specific antigen of the *β*-islet cells of the pancreas that has a p277-like epitope [19]. A study by [20] reported that mouse Hsp60 molecules in *β*-cells of NOD mouse are the target of anti-H-p277. However this finding poses the question of how a ubiquitous molecule such as Hsp60 can be the target of a tissue-specific autoimmune disease? There is no evidence to suggest that tissue-specific Hsp60 exists, given that there is a fibroblast homologue which contains identical sequence to the pancreatic *β*-islet Hsp60 [20]. However, several suggestions have been proposed in order to address this question; Hsp60 is present in a unique way in secretory vesicles of the *β*-cells, and in the event of insulin secretion, these vesicles fuse with the *β*-cell membrane causing Hsp60 to be presented on the *β*-cell membrane and/or even secreted in the absence of mitochondrial stress, causing differences in secretion characteristic from other cells [20, 21]. When C9 cells (a diabetogenic NOD T-cell clone that responds to p277) are injected into NOD mice, they migrate to the pancreas and kidney [20]. Another experiment showed that there are 8–12 genes for Hsp60 in the vertebrate genome; however, when sequenced, all but one were pseudogenes [22]. All of these experiments suggest that although *β*-cell Hsp60 is not tissue-specific, there may be tissue-specific posttranslational modifications of Hsp60 [20]. Experiments have also shown how T-cells are still able to proliferate in the presence of *β*-cell and absence of other antigen-presenting cells suggesting that Hsp60 in *β*-cell may be processed specifically to that tissue, enabling the *β*-cell to present its own Hsp60 to the T-cells, and ultimately present specific immunogenic peptide like p277 [20]. Even though T-cells targets non-tissue-specific molecules, it may have a greater preference over vulnerable, damaged *β*-cells, ultimately causing distress to *β*-cells, and allowing the release of tissue-specific antigens [20]. Expression of Hsp60 in *β*-cell may also be augmented by environmental tissue-specific trigger such as viral infection via *γ*-interferon, thus causing *β*-cells to be targets for anti-Hsp60 T-cells [20].

Thus what is the purpose and what can the immune system gain from recognising Hsp60? Hsp60 is a highly conserved protein; therefore both bacterial (foreign) and endogenous Hsp60 (self) can act as an antigen for *β*-cells [23]. At first, production of antibodies against Hsp60 was thought to be a mechanism to fight bacterial infection or vaccination [23]; however it was subsequently found that autoantibodies against self-Hsp60 were also found to be associated with various autoimmune diseases such as Type 1 diabetes [24].

Various studies with human patients have shown that treatment of Type 1 diabetes with p277 epitope can preserve part of the endogenous insulin production by arresting the destruction of the *β*-cell mass in the pancreas [25, 26]. The paper by Stuart et al., 2012, states that a number of Hsp60 peptide epitopes that can bind multiple allelic variants of the human major histocompatibility complex molecule HLA-DR are called pan-DR epitopes which can induce low peptide-specific proliferative responses and peptide-specific production of intracellular cytokines such as interleukin-10 (IL-10), an anti-inflammatory cytokine, and interferon-*γ* (IFN-*γ*) in Type 1 diabetes [26]. This suggests that Hsp60 and peptides derived from the full-length molecule can induce both proinflammatory and anti-inflammatory cytokines. This confirms Hsp60 as an important modulator of inflammation in Type 1 diabetes mellitus. It should be noted that Hsp60 is also thought to downregulate inflammation via activated effector T-cells upregulating Hsp60 and presenting their own Hsp60 epitopes to antiergotypic regulatory T-cells [27].

Evidence is also accumulating which seem to suggest that Hsp60 may also be involved in the pathogenesis of Type 2 diabetes mellitus. A number of studies have shown elevated levels of Hsp60 protein in systemic circulation in Type 2 diabetes patients. Yuan et al. [10] reported the elevated presence of Hsp60 in both serum and saliva of Type 2 diabetics compared to nondiabetic control subjects. Salivary Hsp60 was found to be fourfold higher in type 2 diabetics compared to nondiabetics, and serum Hsp60 was found to be 16-fold higher than the salivary Hsp60 in Type 2 diabetics. The presence of Hsp60 as a molecular marker that represents mitochondrial stress opens up the opportunity for a noninvasive diagnostic route to further investigate the relationship of Hsp60 and diabetes [10]. Evidence has been documented which shows that when human HeLa cells are grown in the presence of mitochondrial inhibitors (such as sodium azide, hydrogen peroxide, and high glucose) there is a significant upregulation of Hsp60 at the protein level [28]. This suggests that the increased level of serum Hsp60 detected in Type 2 diabetes mellitus patients might also be due to mitochondrial stress.

## 3. Hsp60 and Inflammation

A number of studies suggest that extracellular Hsp60 plays a role as a cellular “danger” signal for cellular and humoral immune reactions [29, 30]. In 1997, a hypothesis was proposed suggesting that elevated acute-phase/stress reactants (such as Hsp60) and their major cytokine are associated with Type 2 diabetes [31]. Since then, many studies have been conducted looking at circulating markers of inflammation, and their association with Type 2 diabetes mellitus [32]. Inflammation has been found to be an important causative factor in the pathogenesis of Type 2 diabetes and insulin resistance [33]. There is also an observable association between the pathogenesis of insulin resistance, diabetes and atherosclerosis, and the activation of innate immune system by toll-like receptors (TLRs) [34–36]. Interestingly, a link between TLR2 and TLR4 polymorphisms and Type 2 diabetes has been documented, suggesting that TLRs may play a causative role in diabetes [37, 38]. Other studies have shown the increased expression of TLR2 and TLR4 in conventional insulin resistance target tissues like skeletal muscle and adipose tissue of Type 2 diabetics [39, 40].

TLRs are a family of protein that senses the invasion of microorganism. This in turn stimulates the TLRs and initiates a range of host defence mechanisms [41]. Each member of the TLR family is set to recognise a specific pathogen component, and when activated it will create a signalling cascade that ultimately leads to the production of cytokines (such as IL-1*β*, IL-6, IL-8, monocyte chemoattractant protein-1 (MCP-1), and tumour necrosis factor-*α* (TNF-*α*)) and adaptive immune response [41]. Ligands for TLR2 and TLR4 include Hsp60, Hsp70, high mobility group B1 protein, endotoxin, hyaluronan, advanced glycation end products, and extracellular matrix components [42]. In 2009, it was postulated that the effects of Hsp60 on the innate immune system may be due to the presence of bacterial contaminants (LPS, lipopolysaccharide, a major cell wall component of gram negative bacteria) in preparations of the recombinant mammalian Hsp60 preparations [43]; however it is now clear that activation of innate immune receptors can be caused by Hsp60 on its own and not by associated contaminants [44]. A study done in 2010 by Dasu et al. confirmed that expression of TLR2 and TLR4 in Type 2 diabetics is greatly increased when compared to nondiabetics. Furthermore, due to the increase of TLR2 and TLR4 expression, there was a subsequent increase of inflammation, which was mediated by nuclear factor Kappa Beta (NF-*κ*B) p65 [45]. The concentrations of proinflammatory mediators IL-1*β*, IL-6, IL-8, MCP-1, and TNF-*α* in the serum were also found to be significantly increased in Type 2 diabetics, compared to nondiabetics. This novel finding suggests that Hsp60 could be playing modulatory responses in inflammation, a metabolic characteristic of Type 2 diabetes, through the activation of TLRs.

Interestingly, antihuman Hsp60 small-hairpin RNAs (shRNAs) have been documented to downregulate the expression of endogenous Hsp60 mRNA 48 hours after transfection in human cells [46]. The study proves that Hsp60 can be regulated using RNAi and opens the possibility to develop RNAi based therapeutic strategies to treat Type 2 diabetes clinically.

Many studies have also shown that people suffering from Type 1 and Type 2 diabetes have accelerated atherosclerosis and are in greater risk of developing atherosclerosis [47]. Atherosclerosis is a disease where plaque builds up inside the arteries and is the cause of a majority of cardiovascular diseases [48]. Early atherosclerosis is characterised by the penetration of agranulocyte or mononuclear cells, in particular monocytes, macrophages, and T-lymphocytes [49]. In the late atherosclerosis lesions, T-lymphocytes were seen to be activated, and a substantial proportion of the cells are thought to be reacting against Hsp60 [50, 51]. A study done using rabbits immunized with mycobacterial Hsp60 have shown that atherosclerotic lesions can be prevented when the rabbit's T-lymphocytes are depleted [52, 53]. On the other hand, when LDL-receptor deficient mice are introduced to the Hsp60 reactive T-lymphocytes, the mice were able to induce pronounced atherosclerotic vessel wall changes [54].

A study done in 2007 found a correlation between atherosclerosis and T-cell reactivity to Hsp60 in young males but not in men aged 50 and above. This suggests that the T-cell reactivity to Hsp60 is more prominent in young and very early stages of arteriosclerosis [55]. It is thought that T-cell reactivity to Hsp60 is less prominent in men age 50 and over because the majority of the T-cells have already formed from blood to the site of inflammation in atherosclerotic plaques and lymphocytes from peripheral blood may no longer present the specific antigen repertoire of cells in vessel walls [55]. This T-cell reactivity to Hsp60 is capable of triggering both innate and adaptive immune responses that initiate the earliest inflammatory stage of atherosclerosis, and mitochondrial Hsp60 is increasingly being recognised as a key autoantigen at the sites of endothelial inflammation [56, 57]. However, the mechanisms leading to expression of Hsp60 during the initiation of arteriosclerosis due to T-cell reactivity to Hsp60 are still not well understood.

## 4. Conclusion

**Figure 1:**
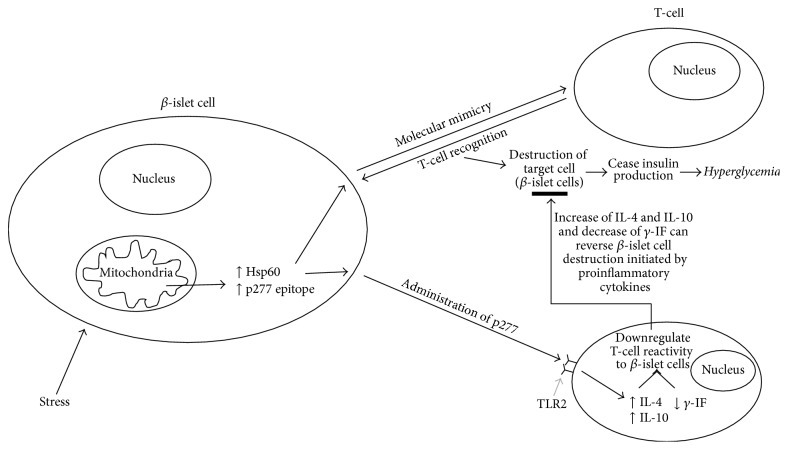
Hsp60 and type 1 diabetes.

**Figure 2:**
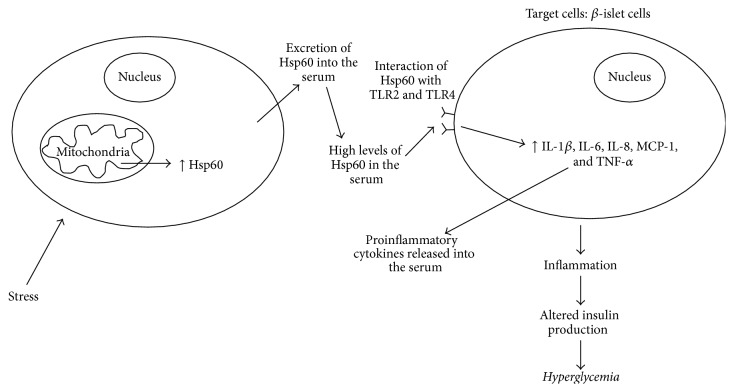
Hsp60 and type 2 diabetes.

There is a clear association between Hsp60 and Type 1 and Type 2 diabetes. In Type 1 diabetes, Hsp60 protein is able to induce the production of anti-Hsp60 antibodies as a defence mechanism against pathogens; anti-Hsp60 antibodies also target endogenous Hsp60 (p277 epitope) and result in the destruction of *β*-islet cells. However, both Hsp60 and p277 peptides can also prevent *β*-cell destruction by upregulation of the anti-inflammatory Th2 cytokine pathway. Since the loss of *β*-islet cells is primarily thought to be driven by a proinflammatory Th1 cytokine response, the shift of Th1 to Th2 by Hsp60 and p277 may be involved in attenuation of Type 1 diabetes mellitus (Figure 1). The high levels of Hsp60 found in the serum in Type 2 diabetic may also lead to the initiation of proinflammatory cytokines in target cells (such as vascular endothelial cells) by interacting with TLR2 and TLR4 receptors (Figure 2). Thus, Hsp60 acting as a proinflammatory signalling molecule may play a role in the nonresolved vascular inflammation, which is increasingly being recognised as a feature of Type 2 diabetes. It is suggesting that Hsp60 does indeed play a key regulatory role in modulating inflammatory processes in diabetes mellitus and could also provide a key link between mitochondrial stress and inflammation in diabetes mellitus.
